# Conception vessel acupuncture research regularity in the treatment of diminished ovarian reserve: a multi-center, large-sample prospective cohort study protocol

**DOI:** 10.3389/fendo.2024.1380444

**Published:** 2024-09-02

**Authors:** Xiaoyu Zhang, Hao Sun, Na Zhang, Zijun Mou, Qingchang Xia, Dongqing Du, Yuxia Ma

**Affiliations:** Department of Acupuncture and Massage College, Shandong University of Traditional Chinese Medicine, Jinan, China

**Keywords:** diminished ovarian reserve, acupuncture, multi-center, conception vessel, study protocol

## Abstract

**Background:**

Diminished ovarian reserve (DOR) refers to a decrease in the number or quality of oocytes in the ovarian cortex, which is a degenerative disease of the reproductive system, and can further develop into premature ovarian failure. There are few studies on acupuncture and moxibustion for DOR, which are still in the exploratory stage.

**Methods/design:**

This study was a real-world case registry study. According to whether the subjects received conception vessel acupuncture or not, they were divided into the basic treatment combined with conception vessel acupuncture group and the basic treatment group. A total of 1221 patients with DOR were enrolled and treated for 12 weeks. The percentage of patients with ≥30% improvement in anti-Müllerian hormone (AMH) was evaluated at the end of week 12. Secondary outcomes included Antral follicle count (AFC), modified Kupperman scale, basal FSH level, LH level, FSH/LH ratio, positive pregnancy, clinical pregnancy, early spontaneous abortion, ongoing pregnancy, and ectopic pregnancy.

**Discussion:**

This study provides clinical evidence and theoretical support for the treatment of DOR with conception vessel acupuncture and moxibustion, so as to guide and improve the efficacy of acupuncture and moxibustion.

**Trial registration:**

Acupuncture-Moxibustion Clinical Trial Registry ChiCTR2400080471. Registered on 30 January 2024.

## Background

Ovarian reserve function reflects the potential of female reproductive endocrine, which depends on the quantity and quality of ovarian reserve follicles, and represents the ability of follicles to grow and develop and form fertilizing oocytes (1). DOR is a risk signal for female fertility decline, which refers to the decrease in the number and/or quality of oocytes in the ovary, accompanied by a decrease in AMH level, a decrease in the number of antral follicles, and an increase in FSH level (2). The clinical incidence of the disease is increasing year by year, and there is a trend of developing to younger age (3, 4). If not treated in time, it will further develop into premature ovarian failure (5, 6). With the extension of childbearing age, the adverse effects of DOR on fertility become more and more serious, and the prevalence of DOR in the population is about 10%-35% (1). The demand for assisted reproduction of women with fertility decline in China will be significantly increased. In order to adapt to the development of society and meet the fertility needs of women with DOR, improving their ovarian function and increasing the clinical pregnancy rate has become a widespread concern in the medical community. Acupuncture and moxibustion has certain advantages in the treatment of DOR. Some studies have found that acupuncture and moxibustion can significantly improve the number of oocytes retrieved and embryo quality, embryo implantation rate and clinical pregnancy rate after 1 to 3 menstrual cycles.

Acupuncture and moxibustion for DOR is still in the exploratory stage. According to the existing experience, the acupoints such as Guanyuan (CV 4), Sanyinjiao (SP 6), Shenshu (BL 23), Zigongxue (EX-CA1) and Zusanli (ST 36).etc are selected. At present, there is no standardized acupuncture-moxibustion diagnosis and treatment plan for the disease. This study will analyze the therapeutic characteristics of the acupoint selection combination of acupuncture and moxibustion for DOR in the real world, and summarize the research on the diagnosis and treatment rules of acupuncture and moxibustion for DOR, so as to provide clinical evidence and theoretical support for the treatment of DOR with acupuncture and moxibustion, and guide to improve the efficacy of acupuncture and moxibustion.

This study is a multi-center, large-sample, prospective study combined with case registration study to summarize the key index data of acupuncture and moxibustion conception vessel in the treatment of DOR, and explore the relevant treatment rules of acupuncture and moxibustion conception vessel in the treatment of DOR.

## Study design and methods

### Objectives

To obtain the clinical efficacy data of acupuncture and moxibustion at conception vessel points or those containing conception vessel points and positive points of conception vessel in the treatment of DOR through a real-world multi-center and large sample registry study, and to reveal the treatment rules of conception vessel on DOR by using multimodal data analysis.

### Study design

**Figure 1:**
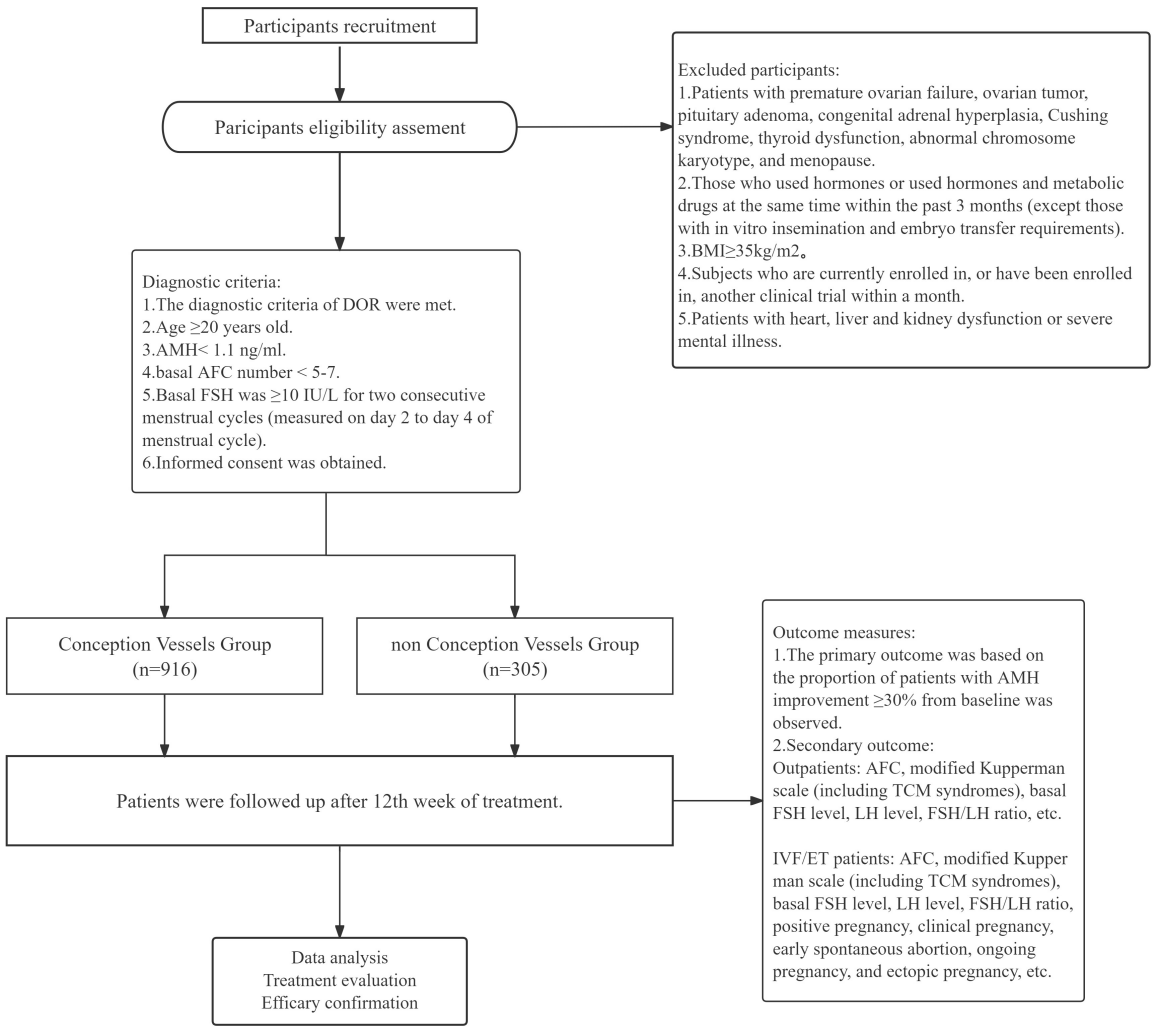
Trial flow chart.

This is a prospective, large sample, multicenter cohort study protocol. This is a real world case registry study and falls under exploratory research. According to whether the subjects received conception vessel acupuncture or not, they were divided into two groups: basic treatment with conception vessel acupuncture group and basic treatment group. A total of 1221 DOR patients from the Affiliated Hospital of Shandong University of Chinese Medicine, the Second Affiliated Hospital of Shandong University of Chinese Medicine and Dongzmen Hospital of Beijing University of Chinese Medicine were included in this study. The percentage of patients with ≥30% improvement in AMH was evaluated at the end of week 12. In addition, AFC, modified Kupperman scale (including TCM syndromes), basal FSH level, LH level and FSH/LH ratio were also observed in outpatients. AFC, modified Kupperman scale (including TCM syndromes), basal FSH level, LH level, FSH/LH ratio, positive pregnancy, clinical pregnancy, early spontaneous abortion, ongoing pregnancy and ectopic pregnancy were also observed in IVF-ET patients. The flow chart and study design schedule are shown in **Figure 1** and **Table 1**, respectively.

**Table 1:** Study schedule of enrolment, intervention, and assessments.

Detection time Target	Screening period	Treatment period			Follow-up period	Pregnancy follow-up (only for pregnant patients)
	0-30 days	4 weeks	8 weeks	12 weeks	16 weeks	
Primary outcome measures						
AMH (day 2-4 of each menstrual cycle)	√	√	√	√	√	
Secondary outcome measures						
Patients in the outpatient clinic						
Modified Kupperman scale (at the end of each menstrual cycle)	√	√	√	√	√	
Antral follicle count (AFC) (days 5-6 of the menstrual cycle before and after treatment)	√				√	
FSH, LH levels, FSH/LH ratio (days 2-4 of each menstrual cycle)	√	√	√	√	√	
Positive pregnancy (urine HCG test at 7-8 days after menstruation, blood HCG test after positive)						√
Clinical pregnancy (gestational sac detected by transvaginal ultrasound 14-21 days after menopause)						√
Early spontaneous abortion (within 12 weeks of gestation)						√
Set of pregnancy indicators for IVF-ET patients						
Ongoing pregnancy (pregnancy lasting 12 weeks or later)						√
Ectopic pregnancy(At 5-6 weeks of menopause, no gestational sac was found in the uterus, pregnancy test was positive, beta - hCG >750Miu/ml)						√
Number of oocytes retrieved (day of oocyte retrieval)						√
Number of normal fertilization (day 2 or 3 after oocyte retrieval)						√
Number of embryos available for transfer and number of high-quality embryos, such as cleavage and blastocyst (3 and 5 days after oocyte retrieval)						√
Rate of mature oocytes or MII oocytes (day of oocyte retrieval)						√
Cycle cancellation rate (number of cycles cancelled/number of cycles initiated ×100%)						√
Embryo implantation rate (implantation 7-10 days after the formation of a fertilized egg, that is, around the third week of the menstrual cycle)						√
Threatened abortion (first 28 weeks of pregnancy)						√
Multiple pregnancy (in the first trimester, after 35 days of menopause)						√
Cumulative pregnancy rate (the percentage of patients who had a successful pregnancy from embryos transferred within a given time or from all embryos transferred for a single oocyte retrieval cycle, as a percentage of all oocytes retrieved cycles)						√

### Ethical approval

This study adheres to the principles of the Declaration of Helsinki. This study has been registered in the Chinese Clinical Trial Registry (Identifier: ChiCTR2400080471, January 30, 2024). The study protocol and informed consent(Version Number: 20231112, V1.0) have been systematically reviewed and approved by the Medical Ethics Committee of the Affiliated Hospital of Shandong University of Traditional Chinese Medicine (Approval Number:2023KY-142, December 26, 2023). All participants will voluntarily sign an informed consent form approved by the ethics committee.

### Participants

A total of 1221 DOR patients were recruited from the Affiliated Hospital of Shandong University of Chinese Medicine, the Second Affiliated Hospital of Shandong University of Chinese Medicine and Dongzhimen Hospital of Beijing University of Chinese Medicine through hospital posters, Wechat, media platforms or online advertisements. Two recruitment staff members at each hospital performed informed-consent consultations in a separate office at the hospital. Participants will be provided with detailed information about the study, including but not limited to the study objectives, interventions, potential benefits, and risks. If participants agreed to participate in the study, they were required to submit a signed informed consent form.

### Diagnostic criteria

DOR is caused by the reduction of the number or quality of oocytes leading to insufficient ovarian function, resulting in decreased fertility, and at the same time, accompanied by the reduction of AMH level or AFC number or the increase of basal FSH level.

At present, there is no gold standard for the diagnosis of DOR in clinical medicine. In this study, according to the "expert consensus on Clinical diagnosis and treatment of diminished Ovarian Reserve" issued by the Reproductive Endocrinology and Fertility Protection Group of the Fertility Protection Branch of the Chinese Preventive Medicine Association in the Journal of Reproductive Medicine, Volume 31 (4) 2022, the following criteria were used:

- AMH<1.1 ng/ml
- Bilateral ovaries: AFC < 5-7
- Basal FSH≥10 IU/L for two consecutive menstrual cycles.
- DOR can be divided into two categories: physiological DOR associated with advanced age and pathological DOR incompatible with age. Women over 35 years old who have been trying for pregnancy for more than 6 months still have no successful pregnancy need to be evaluated for ovarian reserve function.

### Inclusion criteria

- The diagnostic criteria of DOR were met.
- Age ≥20 years old.
- AMH< 1.1 ng/ml.
- basal AFC number < 5-7.
- Basal FSH was ≥10 IU/L for two consecutive menstrual cycles (measured on day 2 to day 4 of menstrual cycle).
- Informed consent was obtained.

### Exclusion criteria

- Patients with premature ovarian failure, ovarian tumor, pituitary adenoma, congenital adrenal hyperplasia, Cushing syndrome, thyroid dysfunction, abnormal chromosome karyotype, and menopause.
- Those who used hormones or used hormones and metabolic drugs at the same time within the past 3 months (except those with *in vitro* insemination and embryo transfer requirements).
- BMI≥35kg/m^2^.
- Subjects who are currently enrolled in, or have been enrolled in, another clinical trial within a month.
- Patients with heart, liver and kidney dysfunction or severe mental illness.

### Stopping and withdrawal criteria

- Subjects withdraw by their own request.
- Subjects were deemed by the investigator to be unfit to continue in the study. For example, they had a serious adverse event, serious complications, or deterioration of their condition requiring a change of treatment.
- Other reasons caused the subjects to withdraw from the trial and lose follow-up.

After the dropout of the subjects, the researchers should contact the subjects as much as possible to ask the reason, record the time of the last treatment, and complete the evaluation items that can be completed. Dropout cases also need to be properly preserved, and their last primary efficacy indicators should be transferred to the final outcome for statistical analysis.

### Interventions

#### Screening period

During the screening period, physicians collected the baseline data of the subjects, including basic medical history, general information, etc., and evaluated the subjects' data according to the inclusion and exclusion criteria. According to the evaluation, the subjects were determined to meet the inclusion criteria, and signed the informed consent.

#### Period of treatment

Doctors selected treatment regimens according to the actual conditions of the subjects, including basic treatment, ordinary acupuncture, electroacupuncture and moxibustion. The acupuncture prescriptions of the subjects were recorded whether the conception vessel acupuncture method, outcome indicators and adverse events were included in the acupuncture prescriptions.

#### Basic treatment

According to the clinical practice of each participating hospital, the basic treatment was traditional Chinese medicine (such as "Erzhi-Tiangui-Decoction").

#### Recommended acupoints

**Figure 2:**
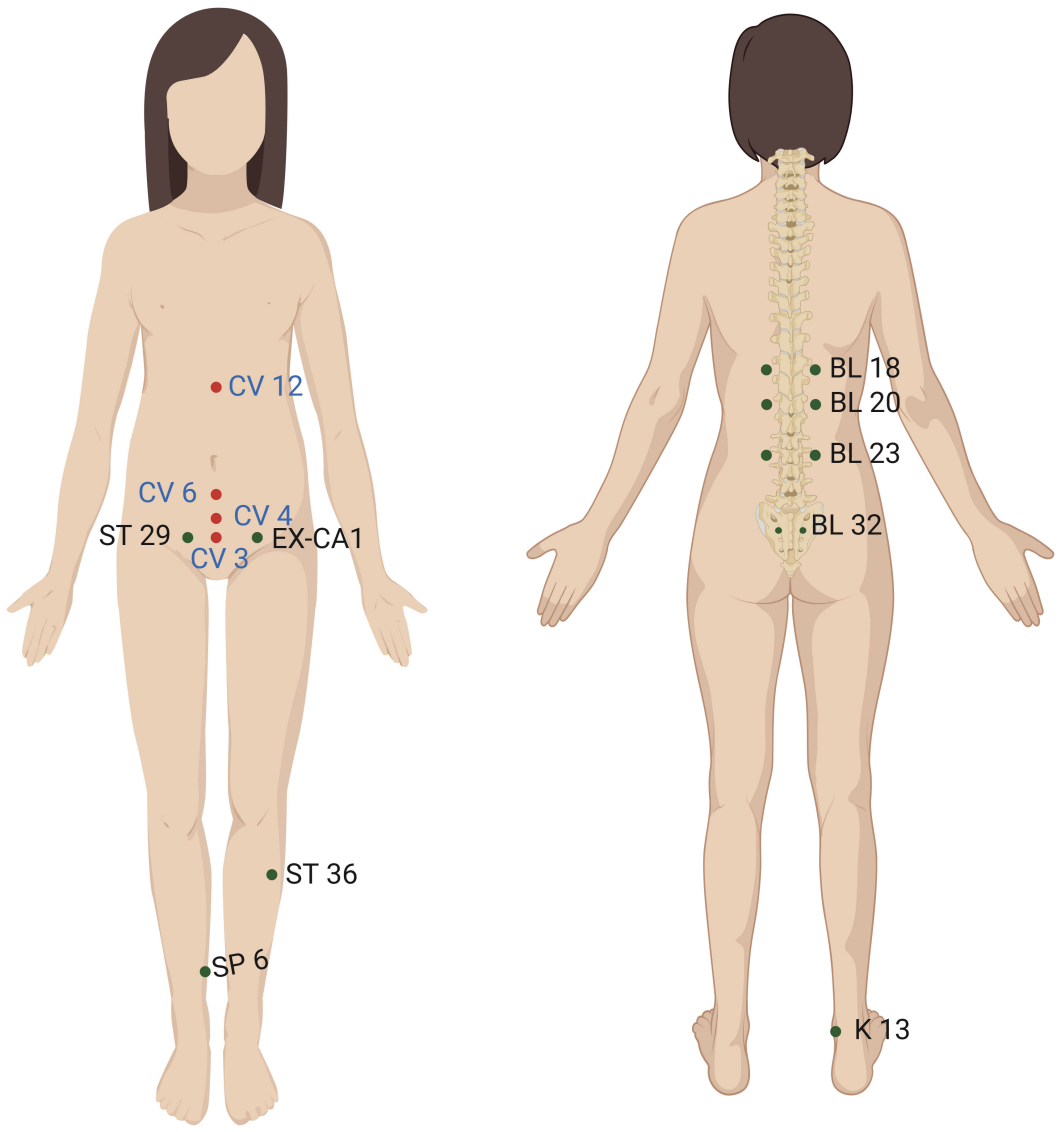
The location of the acupoints on the body surface.

The acupoints selected included ST 36(Zusanli), SP 6(Sanyinjiao), CV 4(Guanyuan), CV 6(Qihai), CV 3(Zhongji), CV 12(Zhongwan), K13(Taixi), BL 23(Shenshu), BL 18(Ganshu), BL 20(Pishu), EX-CA1(Zigongxue), ST 29(Guilai), BL 32(Ciliao) and conception vessel positive reaction points (the positive reaction points were selected from the previous examination of the patient's conception vessel by the meridian diagnostic instrument) (**Figure 2**; **Table 2**). The group with conception vessel positive reaction points, CV 6(Qihai), CV 3(Zhongji), CV 12 (Zhongwan) and CV 4(Guanyuan) was the conception vessel group. Doctors select recommended acupuncture points based on the patient's condition, adjusting needle insertion depth and adding or reducing points according to symptoms and individual differences. For more details on treatment cycles, techniques, and frequency of acupuncture and moxibustion, please refer to the section on Ordinary acupuncture methods and treatment courses.

**Table 2:** Acupoint location.

Acupoint	Location	Insertion depth
CV4(Guanyuan)	On the mid-line of the abdomen, 3/5 of the way down from the umbilicus to the superior edge of the pubic bone.	3-3.5 cun
SP6(Sanyinjiao)	3 cun directly above the tip of the medial malleolus, on the posterior border of the tibia.	1-1.5 cun
CV3(Zhongji)	On the midline of the lower abdomen, 4 cun below the umbilicus.	1-1.2 cun
CV6(Qihai)	On the anterior midline of the lower abdomen, 1.5 cun below the umbilicus.	1-1.5 cun
BL32(Ciliao)	The midpoint of the line connecting Pangguangshu and the lower border of the spinous process of the 2nd sacral vertebra.	1-2 cun
CV12 (Zhongwan)	On the anterior midline of the abdomen, 4 cun above the umbilicus.	1-1.5 cun
K13(Taixi)	In the depression between the prominence of the medial malleolus and heel tendon.	0.5-0.8 cun
ST36(Zusanli)	3 cun below Dubi, one finger-breadth from the anterior crest of the tibia.	1-2 cun
BL23(Shenshu)	1.5 cun lateral to the lower border of the spinous process of the 2th lumbar vertebra.	0.5~1cun
BL18(Ganshu)	Puncture perpendicularly or a bit obliquely in the direction of the midline 0.5-0.8 cun.	0.5-0.8 cun
BL20(Pishu)	Puncture perpendicularly or a bit obliquely in the direction of midline 0.5-0.8cun.	1.5 cun
EX-CA1(Zigongxue)	On the lower abdomen, 4 cun below the umbilicus, 3 cun lateral to CV3	1.5-2 cun
ST29(Guilai)	Lower abdomen, 4 cun below the umbilicus, 2 cun lateral to the midline.	1.5 cun

### Ordinary acupuncture methods and treatment courses

#### Treatment duration and frequency

1) Ordinary acupuncture and electroacupuncture were applied once every other day, avoiding the menstrual period. Regular menstrual cycle was a course of treatment, irregular menstrual cycle was 4 weeks as a course of treatment. The intervention was given for 3 menstrual cycles (or 12 weeks). The efficacy was observed after each course of treatment, and at least 1 menstrual cycle was followed up after 3 courses.
2) The frequency of treatment was determined by doctors according to the type of moxibustion and the actual condition of patients. Regular menstrual cycle was a course of treatment, irregular menstrual cycle was 4 weeks as a course of treatment. The intervention was given for 3 menstrual cycles (or 12 weeks). The efficacy was observed after each course of treatment, and at least 1 menstrual cycle was followed up after 3 courses.

#### Methods of treatment

1) Ordinary acupuncture

Different specifications of Hwato brand disposable sterile acupuncture needles (0.30mm×40mm or 0.30mm×50mm) and SDZ-III electro-acupuncture instrument (Medical Equipment, Suzhou, Jiangsu, China) will be used in this study.

Description of reinforcing and reducing techniques: after acupuncture at Deqi, flat reinforcing and flat reducing techniques were applied to each acu point for about 0.5min. Thereafter, acupuncture was applied once every 10min, about 0.5min each time, and the needles were left for 30min. In the multi-acupoint group, the order of acupuncture was carried out according to the principle of "Yang first, Yin later, up first and down later".

2) Electroacupuncture

Acupuncture operation: Acupuncture operation is the same as ordinary acupuncture.

Electroacupuncture operation: the electroacupuncture instrument is connected to the acupuncture needle to ensure the connection is stable, and the appropriate electroacupuncture parameters, such as frequency and intensity, are set.

Electroacupuncture stimulation: the intensity of the current stimulation was gradually increased, so that the subject could feel the slight stimulation but did not cause pain.

At the end of the treatment, the current stimulation was stopped after 30min of continuous EA stimulation, the wire was pulled out first, and then the needle was slowly removed.

3) Moxibustion

Prepare moxa cones, moxa sticks or pressed moxa cones and moxa cone appliances, such as moxa cone box, moxa cone frame or moxa cone moxibustion box, and choose the appropriate moxibustion scheme. The moxibustion time of each acupoint or positive reaction point was controlled, generally 15-30 minutes.

If necessary, the separated moxibustion can be used: the appropriate materials (such as ginger flakes, medicinal powder, etc.) are placed between the moxa cone appliance and the skin, and then the lit moxa sticks, moxa sticks, or pressed moxa cones are placed above. Ensure adequate isolation between the material and the skin to avoid scalding.

If necessary, warm acupuncture can be used: after selecting the acupoint plan, acupuncture can be performed, and moxa sticks or moxa cones are fixed on the handle of the needle and ignited. Pay attention to control the temperature and time to ensure safety and comfort.

As needed, mild moxibustion can be used: the lit moxa sticks or pressed moxa cones can be aimed at the acupoints at an appropriate distance from the skin to avoid scalding.

#### Concomitant treatments

Basic treatment, ordinary acupuncture, electroacupuncture and moxibustion were the basic treatments in this study. During the treatment process, clinical practitioners may combine the above basic therapies based on clinical practice and meticulously document the treatment details in case report forms. Such as traditional Chinese medicine (medication time, syndrome differentiation, treatment principle, prescription), western medicine treatment (drug name, dosage, unit, frequency of administration, route of administration, duration of inches, indications).

### Outcome measures

#### Primary outcome measures

At the end of the 12th week of treatment, the proportion of patients with AMH improvement ≥30% from baseline was observed. AMH is the most accurate biomarker of ovarian aging (7). Compared with other hormones, AMH can reflect the decline of ovarian reserve with age earlier, and its level is not affected by menstrual cycle, hormonal contraceptives and pregnancy. AMH detection can make up for the deficiency of traditional hormone detection in evaluating ovarian reserve function, and can evaluate ovarian reserve function reliably and quickly.

### Secondary outcome measures

#### Outpatients

AFC, modified Kupperman scale (including TCM syndromes), basal FSH level, LH level, FSH/LH ratio, positive pregnancy, clinical pregnancy, early spontaneous abortion, ongoing pregnancy, ectopic pregnancy, etc.

#### IVF-ET patients

AFC, modified Kupperman scale (including TCM syndromes), basal FSH level, LH level, FSH/LH ratio, positive pregnancy, clinical pregnancy, early spontaneous abortion, ongoing pregnancy, and ectopic pregnancy; Embryo implantation rate, number of oocytes retrieved, number of normal fertilization, number of transferable embryos and high quality embryos, mature oocytes rate or MII oocytes rate, cancellation cycle rate, threatened abortion, multiple pregnancy rate, cumulative pregnancy rate.

The above outcome measures were measured during the screening period, at the end of the menstrual cycle after each treatment, and during the follow-up period.

#### Adverse events

Adverse events are unsafe risks, states or negative events with consequences caused by various factors other than the natural course of the subject's own disease, which are actively discovered by the staff in the medical institution or appear in the process of the subject's receiving medical services. For example, acupuncture caused dizzy, needle stagnation, subcutaneous hemorrhage, hematoma, moxibustion caused burns, red rash, after acupuncture treatment, and other adverse reactions.

### Criteria for determining efficacy

According to the "Guiding Principles for Clinical Research of New Chinese Medicine" (2002 edition), the efficacy criteria of DOR were determined:

Recovery: pregnancy, or ≥70% AMH improved from baseline

Significant effect: 50%≤AMH improved from baseline <70%。

Effective: 30%≤AMH improved from baseline <50%

No effect: AMH improved from baseline <30%。

### Adherence assessment

The sessions of treatments will be recorded in the CRF to assess the adherence of patients.

### Statistical considerations

#### Sample size

This study is a prospective cohort registration study design. The Conception Vessel Acupuncture group consists of patients receiving Conception Vessel Acupuncture treatment in addition to basic treatment, while the basic treatment group includes patients receiving only traditional Chinese medicine treatment or ovulation induction protocols. Based on literature review and clinical expert experience, the expected improvement rate is 60% for the Conception Vessel Acupuncture group and 50% for the basic treatment group. Set a two-sided significance level of 0.05 with a statistical power of 80%. According to clinical practice and patient treatment preferences, the ratio of the two groups is set at 3:1.

The sample size calculation formula:


$$n_{1}=kn_{2}$$

$$n_{2}=\frac{{\left(Z_{1-\alpha /2}+Z_{1-\beta }\right)}^{2}}{{\left(P_{1}-P_{2}\right)}^{2}}\left[\frac{P_{1}\left(1-P_{1}\right)}{K}+P_{2}\left(1-P_{2}\right)\right]$$

Based on this formula, it was calculated that the Conception Vessel Acupuncture group requires 259 subjects and the basic treatment group requires 778 subjects (rounded up). Considering a dropout rate of 15%, the final required sample size is 916 for the Conception Vessel Acupuncture group and 305 for the basic treatment group, totaling 1221 subjects to be enrolled in the study.

### Statistical analysis

The study data set was statistically analyzed against the per-protocol (PP) analysis set. PP set mainly refers to the cases with good compliance, protocol compliance, and completion of all the important information required for the registration study, while the cases with poor compliance, not in line with the study protocol, or missing important information were excluded from the analysis. The continuous variables with normal distribution were expressed as ± s, the non-normal distribution data were expressed as median and quartile, and the categorical data were expressed as constituent ratio or percentage. The independent sample t test and analysis of variance were used for the comparison of continuous variables between groups, and the rank sum test was used for non-parametric data comparison. Categorical variables were compared using the chi-square test or Fisher's exact test. Missing data were imputed by multiple imputation using chained equations (mice package in R language) to generate 5 complete data sets. Additionally, logistic regression modeling was applied to explore the association between predictor variables and the outcome of interest, adjusting for potential confounders identified during the analysis. Odds ratios (ORs) were calculated from the logistic regression models to quantify the strength and direction of the association between each predictor variable and the outcome. Odds ratios provide a measure of the likelihood of the outcome occurring in one group compared to another, based on the presence or absence of the predictor variable.All data analysis and modeling were performed using R3.4.4 software. P < 0.05 was considered significant.

### Data management

#### Skill and experience of treating physicians

To minimize protocol deviations, treating physicians were rigorously trained and assessed before the start of the study to ensure that they had adequate expertise and skills;

#### External intervention control

During a clinical trial, subjects may receive other non-study related interventions such as medication or other alternative therapies. No other interventions were specified, monitored, and recorded before the start of the trial.

#### Hypothesis testing and permutation testing

Hypothesis testing methods were used to evaluate the main results of the study and to determine significant differences. To further verify the reliability of the results, a permutation test was used to rearrange the original data and repeat the statistics.

#### Confounding factor control

Through randomization stratification, paired design, multiple regression analysis, other factors that may interfere with the results can be controlled and adjusted.

#### Data monitoring and quality control

A strict data management and monitoring system was established, including data collection, data entry, data storage and data cleaning to ensure the accuracy and integrity of data.

### Quality control

The real-world clinical research of acupuncture and moxibustion should also follow clear and effective quality control methods to ensure the integrity, accuracy and timeliness of clinical data. Investigators should perform their own duties, strictly follow the clinical trial protocol, and adopt standard operating procedures to ensure the implementation of the quality control and quality assurance system of clinical trials. All relevant observations and findings in clinical trials should be verified, and quality control must be carried out at every stage of data processing to ensure that the data are complete, accurate, true and reliable. This program adopts a three-level quality control system of internal quality control, monitoring and inspection. First, internal quality control conducted a monthly self-examination during the treatment phase and a monthly self-examination during the follow-up phase, and a self-examination checklist was completed and submitted. Second, the person in charge of the project appoints the personnel of the undertaking unit to carry out irregular inspections of the research activities, data collection, records and reports of the collaborating unit, which can be carried out online and offline. Third. The principal investigator may entrust auditors (persons not directly involved in the clinical trial) to conduct a systematic examination of the trial-related activities and documents to evaluate whether the trial was conducted in accordance with the trial protocol, standard operating procedures and relevant regulatory requirements, and whether the trial data were recorded in a timely, true, accurate and complete manner.

### Safety assessment

In order to ensure the safety of the study protocol, the incidence of adverse events is usually calculated in advance using the use of adverse event rate = (total number of adverse events/overall sample size) * 100. During the course of the protocol, each adverse event was described in detail, including the type of event, severity, possible cause, and management. For specific adverse events, statistical methods can be used to assess their association with the acupuncture intervention, using the chi-square test or Fisher's exact test to compare different intervention groups. In addition, the protocol should specify requirements for safety reporting, including the documentation of adverse events, results of statistical analyses, and possible safety concerns.

### Protocol amendments

Any protocol modification that could affect study conduct, potential subject benefit, or subject safety, including changes in study objectives, study design, patient population, sample size, or study procedures, would require formal protocol modification. Such revisions would be agreed to by the DMC and submitted to the ethics advisor and relevant local ethics review bodies.

## Discussion

DOR has become a common gynecological disease, mainly characterized by oligomenorrhea, amenorrhea, fertility decline, accompanied by low estrogen and high gonadotropin performance (8). With the development of The Times, the social pressure is increasing, and the incidence of ovarian insufficiency is gradually increasing and younger age, which seriously affects the mental and physical health of women (9). At present, it has become a key concern in the medical field. Early treatment through lifestyle intervention, exogenous hormone supplementation and immunosuppression can effectively improve the clinical symptoms and gonadal hormone levels. If the disease is further developed to a more severe degree, it is difficult for ordinary drugs to work, especially for patients with fertility requirements who may need ovarian transplantation or assisted reproductive technology to obtain pregnancy (10).

A large number of studies have proved that acupuncture has multi-level and multi-target advantages in the treatment of DOR (11–13). Acupuncture can regulate the function of reproductive endocrine system and improve the level of sex hormones (14, 15). It can restore the normal morphology of ovarian tissue cells and provide a favorable environment for the growth and development of follicles (16). It inhibits granulosa cell apoptosis, promotes follicle development and maturation, and reduces follicle atresia (17). It can regulate the expression level of signal molecules in various signaling pathways and improve the ovarian secretion and synthesis function (18). It can improve the rate of ovulation and fertilization in the process of assisted reproduction, and improve the clinical pregnancy outcome (17).

Currently, there is a wealth of randomized controlled trials and meta-analyses investigating the effects of acupuncture on ovarian function. However, existing research predominantly focuses on polycystic ovary syndrome (PCOS) and premature ovarian insufficiency (POI), with limited studies on DOR (19–23). And there are some shortcomings in the existing studies, such as the sample size is generally small, the results are not representative, and many clinical studies do not follow the principle of syndrome differentiation and treatment of traditional Chinese medicine for acupoint selection in acupuncture and moxibustion treatment. Additionally, There is a lack of unified standards for acupoint stimulation amount, needle retention time and treatment cycle, which is difficult to interpret the phenomenon and law from the existing results, and is not conducive to clinical application and promotion. our research specifically targets the impact of conception vessel acupuncture on DOR. This study utilizes a large-sample prospective cohort design to analyze and explore the selection and prescription of acupuncture points in real-world settings, aiming to reveal specific treatment patterns of Conception Vessel Acupuncture for DOR. The study aims to validate traditional theories such as the "meridian-viscera association" and specific “pulse diagnosis-ovary association” theories based on clinical evidence, developing optimized acupuncture treatment protocols for DOR.

Through this multi-center, large-sample prospective cohort study, our team aims to analyze and explore the efficacy characteristics of acupoint selection and prescription in the real-world treatment of DOR with acupuncture and moxibustion, summarize the rules of treating DOR with acupuncture and moxibustion at conception pulse, provide clinical evidence and theoretical support for the treatment of DOR with acupuncture and moxibustion, and guide to improve the efficacy of acupuncture and moxibustion.

### Trial status

Patient recruitment for this trial began on January 31, 2024 and is expected to be completed by December 31, 2024, with data analysis to be completed by January 31, 2025.
